# Supplementary material for: Identification and functional analysis of non-coding regulatory small RNA FenSr3 in Bacillus amyloliquefaciens LPB-18
Source: PeerJ. 2023 May 15;11:e15236. doi: 10.7717/peerj.15236 (PMC10194069; doi:10.7717/peerj.15236)
Supplement: Supplemental Information 4 [file peerj-11-15236-s004.zip › KO/CK-vs-T1_map/map05012.html]

KEGG PATHWAY: Parkinson disease - Reference pathway


|  |  |
| --- | --- |
| **Parkinson disease - Reference pathway** |  |

[
Pathway menu
| Organism menu
| Pathway entry
| Show description
| User data mapping
]

Parkinson disease (PD) is a progressive neurodegenerative movement disorder that results primarily from the death of dopaminergic (DA) neurons in the substantia nigra pars compacta (SNc). Mutations in alpha-synuclein, UCHL1 (a ubiquitin carboxy-terminal hydrolase L1), parkin, DJ1 (a parkin-associated protein involved with oxidative stress), and PINK1 (a putative serine threonine kinase) are known to cause early-onset PD. Mutations or altered expression of these proteins contributes to the damage and subsequent loss of DA neurons through common mechanisms that result in proteasome dysfunction, mitochondrial impairment, and oxidative stress. The demise of DA neurons located in the SNc leads to a drop in the dopaminergic input to the striatum. This results in a reduced activation of the direct pathway and in a disinhibition of the indirect pathway, which is associated with the elevation of adenosine A2A receptor transmission. Such unbalanced activity of the striatal output pathway is at the basis of the motor impairment observed in PD.

|  |  |  |
| --- | --- | --- |
| Reference pathway | 184% 150% 122% 100% 82% 67% 55% | 图片下载 |
